# Toyocamycin attenuates free fatty acid-induced hepatic steatosis and apoptosis in cultured hepatocytes and ameliorates nonalcoholic fatty liver disease in mice

**DOI:** 10.1371/journal.pone.0170591

**Published:** 2017-03-09

**Authors:** Ikuko Takahara, Yuko Akazawa, Maiko Tabuchi, Katsuya Matsuda, Hisamitsu Miyaaki, Youko Kido, Yasuko Kanda, Naota Taura, Ken Ohnita, Fuminao Takeshima, Yusuke Sakai, Susumu Eguchi, Masahiro Nakashima, Kazuhiko Nakao

**Affiliations:** 1 Division of Gastroenterology and Hepatology, Nagasaki University School of Medicine, Nagasaki, Japan; 2 Department of Tumor and Diagnostic Pathology, Atomic Bomb Disease Institute, Nagasaki University, Nagasaki, Japan; 3 Department of Surgery, Nagasaki University, Graduate School of Biomedical Sciences, Nagasaki, Japan; University of Leeds, Faculty of Medicine and Health, UNITED KINGDOM

## Abstract

**Background and aims:**

A high serum level of saturated free fatty acids (FFAs) is associated with the development of nonalcoholic fatty liver disease (NAFLD). X-box binding protein-1 (XBP-1) is activated by FFA treatment upon splicing. XBP-1 is a transcription factor induced by the endoplasmic reticulum (ER) stress sensor endoribonuclease inositol-requiring enzyme 1 alpha (IRE1α). However, the role of XBP-1 in NAFLD remains relatively unexplored. Toyocamycin was recently reported to attenuate the activation of XBP-1, possibly by inducing a conformational change in IRE1α. In this study, we examined the effect of toyocamycin on hepatocyte lipoapoptosis and steatosis. We also explored the effects of toyocamycin in a mouse model of NAFLD.

**Methods:**

Huh-7 cells and isolated rat primary hepatocytes were treated with palmitic acid (PA), which is a saturated FFA, in the presence or absence of toyocamycin. In addition, male C57BL/6J mice were fed a diet rich in saturated fat, fructose, and cholesterol (FFC) for 4 months, after which the effect of toyocamycin was assessed.

**Results:**

Toyocamycin attenuated FFA-induced steatosis. It also significantly reduced PA-induced hepatocyte lipoapoptosis. In addition, toyocamycin reduced the expression of cytosine-cytosine-adenosine-adenosine-thymidine enhancer-binding protein homologous protein (CHOP), which is a key player in ER stress-mediated apoptosis, as well as its downstream cell death modulator, death receptor 5. In the *in vivo* study, toyocamycin ameliorated the liver injury caused by FFC-induced NAFLD. It also reduced hepatic steatosis and the expression of lipogenic genes.

**Conclusions:**

The data we obtained suggest that toyocamycin attenuates hepatocyte lipogenesis and ameliorates NAFLD *in vivo* and may therefore be beneficial in the treatment of NAFLD in humans.

## Introduction

Nonalcoholic fatty liver disease (NAFLD) is a common liver disease that has become a major concern worldwide. NAFLD is a hepatic manifestation of metabolic syndrome and includes liver disorders ranging from simple steatosis to nonalcoholic steatohepatitis (NASH) with liver dysfunction [1]. Long-term follow-up studies in patients with NAFLD suggest that, once NASH develops, it causes cirrhosis and can lead to end-stage liver disease or hepatocellular carcinoma [2]. However, currently, there is no available standard pharmacotherapy for NAFLD.

Increase in the serum level of free fatty acids (FFAs) is associated with the development of NAFLD. The mechanism is associated with insulin resistance, which is a feature of metabolic syndrome [3]. Under healthy conditions, FFAs in hepatocytes are esterified into neutral lipids within the endoplasmic reticulum (ER). However, excess FFA levels disturb ER function and lead to an ER stress response that induces cell death via mitochondrial dysfunction [4, 5]. Key transducers of the ER stress include protein kinase R-like ER kinase (PERK), serine/threonine-protein kinase endoribonuclease inositol-requiring enzyme 1 (IRE1α), and activating transcription factor 6 (ATF6), which are located in the ER lumen. Activation of PERK induces the expression of the transcription factor cytosine-cytosine-adenosine-adenosine-thymidine enhancer-binding protein homologous protein (CHOP), which is a key player in hepatocyte lipotoxicity [6]. In contrast, activation of IRE1α induces the activation of c-Jun N-terminal kinase (JNK) by X-box binding protein-1 (XBP-1), which is a member of the cAMP response element-binding protein/activating transcription factor (ATF) family of transcription factors. However, JNK activation during lipoapoptosis is most likely independent of ER stress. Rather, it is possibly mediated by serine/threonine kinase-, glycogen synthase kinase-3 (GSK3) beta-, and mixed-lineage kinase 3 (MLK3)-dependent mechanisms [7–9]. The abovementioned pathways merge to induce mitochondrial dysfunction, which is mediated by proapoptotic BH3-only proteins, especially Bcl-2-like protein 11 (Bim) [10–12]. Bim activates the proapoptotic Bcl-2 protein Bax in the mitochondria to release cytochrome *C* into the cytosol, which in turn initiates a downstream caspase program leading to apoptosis [4, 12].

ER stress increases the amount of total XBP-1 mRNA via ATF6 activation [13]. XBP-1 mRNA is then spliced into its active form upon a conformational change in IRE1α, which occurs after the phosphorylation of IRE1α. Thus, induction of total XBP-1 mRNA and its splicing are probably essential for triggering XBP-1 activation [13]. It is generally accepted that the IRE1α/XBP-1 pathway contributes to organized folding and degradation of proteins in the ER [14]. Although XBP-1 is crucial for cellular development and the survival of several types of malignant cells, its role differs depending on the organ and cell type in which it is found among other conditions [15–18]. A recent study suggested that during FFA elevation in serum, ER stress response may be directly activated by membrane lipid saturation and not only by unfolded proteins [19]. More importantly, a recent study suggested that XBP-1 is required for *de novo* lipogenesis in the liver [15]. The role of XBP-1 in NAFLD/NASH has been a source of controversy. One study showed that systemic deletion of XBP-1 by siRNA in a mouse model ameliorated NAFLD [20]. Conversely, a lack of hepatic XBP-1 has been reported to result in increases in ER stress and sensitivity to liver injuries including NASH [21, 22].

Toyocamycin was recently reported to attenuate the activation of XBP-1, possibly by inhibiting XBP-1 splicing via a conformational change in IRE1α [23]. Because toyocamycin does not affect the autophosphorylation of IRE1α, it does not inhibit ER stress-induced JNK activation [23]. The effect of the pharmacological inhibition of XBP-1 in NAFLD/NASH is largely unexplored. In the current study, we sought to investigate the effect of toyocamycin in FFA-treated hepatocytes as well as in a mouse model of NAFLD.

## Materials and methods

### Cell culture

Huh-7 human hepatocellular carcinoma cells (#JCRV0403; NIBIOHN, Osaka, Japan) and rat primary hepatocytes were used for the *in vitro* study. Huh-7 cells were cultured in Dulbecco's Modified Eagle medium (DMEM) supplemented with 10% (v/v) fetal bovine serum (FBS) and 1% penicillin/streptomycin as previously described [4]. Rat primary hepatocytes were cultured in Hepato-STIM^TM^ Hepatocyte Defined Medium with 5 μg of epidermal growth factor (provided per 500 mL of medium; BD Biosciences, Bedford, MA, USA) and 200 mM L-glutamine (Wako Pure Chemical Industries, Ltd., Tokyo, Japan).

### Isolation of rat primary hepatocytes

Male Wistar rats weighing 250 g were obtained from Kyudo Co. Ltd. (Saga, Japan). The rats were housed in a temperature-controlled environment under a 12 h/12 h day/night cycle and with free access to standard rat chow and water. All experiments were performed according to the Guidelines for Animal Experimentation at Nagasaki University. Cells were isolated by a modified collagenase perfusion method from the rats as originally described by Berry and Friend [24]. After liver dissociation, the cells were filtered through a 45-μm cell strainer and washed three times at 50 g for 2 min. The cells were then suspended in 40% Percoll Plus solution (GE Healthcare, Tokyo, Japan) and centrifuged at 50 g for 20 min at 4°C to further purify the hepatocytes and enrich the viable cells. Cell viability was determined by trypan blue dye exclusion staining. Hepatocytes with > 80% viability were used for experiments.

### Fatty acid treatment

Palmitic acid (PA) and oleic acid (OA) were purchased from Sigma-Aldrich (St. Louis, MO, USA). The FFAs were dissolved in isopropyl alcohol at concentrations ranging from 20 to 80 mM. They were added to DMEM containing 1% bovine serum albumin to ensure a physiological ratio between the bound and unbound FFAs in the medium [4, 11]. The concentration of FFAs used in the study ranged from 200 to 800 μM, which is similar to the fasting plasma total FFA concentration observed in human nonalcoholic steatohepatitis.

### Quantification of apoptosis

Apoptosis was quantified using the nuclear binding dye 4′,6-diamidino-2′-phenylindole dihydrochloride (DAPI) (Sigma-Aldrich) and a fluorescence microscope (BZ-9000; Keyence, Osaka, Japan). Huh-7 cells were stained with 5 μg/mL DAPI at 37°C for 30 min. Apoptotic cells were measured by counting 100 random cells per study. Apoptosis was expressed as a percentage of the total cells counted [11] and was confirmed biochemically by the caspase-3/7 activation assay. For the assay, the cells were initially cultured in 96-well plates, after which caspase-3/7 activity was measured using Apo-ONE^®^ Homogeneous Caspase-3/7 kit (Promega, Madison, WI, USA) according to the manufacturer’s instructions.

### Nile red staining

Huh-7 cells were cultured on Nunc^®^ Lab-Tek^®^ II Chamber Slides^TM^ (Sigma-Aldrich) and treated with a combination of PA (200 μM) and OA (200 μM) in the presence or absence of toyocamycin (Sigma-Aldrich). The above combination of the FFAs was employed because treatment with a combination of saturated and unsaturated FFAs enhances steatosis more than treatment with a saturated FFA alone does, which is optimal in steatosis assays [4]. Intracellular neutral lipids were stained with 1 μg/mL Nile Red (Sigma-Aldrich) at 25°C for 5 min. The cells were fixed with 3.7% paraformaldehyde at 37°C for 15 min, after which they were washed twice with phosphate-buffered saline (PBS). The cells were then mounted using ProLong^®^ Gold Antifade Reagent with DAPI and visualized under an inverted laser-scanning confocal microscope at excitation and emission wavelengths of 577 and 590 nm, respectively.

### Real-time polymerase chain reaction (PCR)

Total RNA was extracted from the cells using TRIzol reagent (Invitrogen, Waltham, MA, USA) and reverse-transcribed into cDNA with Moloney murine leukemia virus reverse transcriptase (Invitrogen) and random primers (Invitrogen). For real-time PCR analysis, cDNA samples were amplified using SYBR Green (Molecular Probes, Eugene, OR, USA). The primers *5′-AGTGGGTATTTCTCTTTTGACACAG-3′* and *5′-GTCTCCAATACGCCGCAACT-3′* were used to amplify human Bim, *5′-AGTGGGTATTTCTCTTTTGACACAG-3′* and *5′-GTCTCCAATACGCCGCAACT-3*′ were used for human DR5, and forward *5′-ATGGCAGCTGAGTCATTGCCTTTC-3′* and reverse *5′-AGAAGCAGGGTCAAGAGTGGTGAA-3′* were used for CHOP.

Primers for 18S rRNA (Ambion, Austin, TX, USA) were used as the internal control. The relative mRNA expression levels were expressed as a ratio of target mRNA/18S rRNA for each sample.

### Detection of XBP-1 splicing

Huh-7 cells were treated with either vehicle (Veh), 800 μM PA, 1 μM toyocamycin (Sigma), or 800 μM PA + 1 μM toyocamycin for 8 h. RNA was then extracted and converted to cDNA. XBP-1 cDNA encompassing the region of the *Pst*I restriction site was amplified using the following primers: forward primer, *5'-AAACAGAGTAGCAGCTCAGACTGC-3'* and reverse primer, *5'-TCCTTCTGGGTAGACCTCTGGGAG-3'*. Samples were subjected to electrophoresis on 0.8% agarose gels. The gels were then photographed under UV illumination. In the vehicle-treated cells, majority of Xbp-1 PCR products were unspliced and cut by *Pst*I, producing the native unspliced form of *Xbp-1* mRNA (Xbp-1u with a size of 474 bp) that is sensitive to *Pst*I digestion, which resulted in 285- and 189-bp products. A 448-bp amplification product, indicative of spliced *Xbp-1* mRNA (Xbp-1s), was not sensitive to *Pst*I digestion because the *Pst*I site was spliced out and the 448-bp band was increased in the PA-treated cells.

### Immunoblot analysis

Protein extracts from the cells were prepared by washing the cells in PBS and incubating them in ice-cold lysis buffer supplemented with a protease inhibitor cocktail. After triple sonication and evaluation of the protein concentration, the protein samples (60 μg/lane) were subjected to 4–15% sodium dodecyl sulfate-polyacrylamide gel electrophoresis and electrotransferred to a nitrocellulose membrane for treatment with specific antibodies. Blots were developed using the alkaline phosphatase colorimetric system. The following primary antibodies were used: rabbit-anti Bim, rabbit-anti phospho-SAPK/JNK, rabbit-anti SAPK/JNK, rabbit-anti cleaved PARP, and rabbit-anti PARP. The antibodies were purchased from Cell Signaling Technology Inc. (Danvers, MA, USA). Goat-anti actin antibodies were purchased from Santa Cruz Biotechnology (Santa Cruz, CA, USA). Proteins were detected using horseradish peroxidase-conjugated rabbit anti-goat or goat anti-rabbit secondary antibodies (Life Technologies, Inc., Gaithersburg, MD, USA). The bound antibodies were visualized using a chemiluminescent substrate (SuperSignal^®^ West Pico Chemiluminescent Substrate; Thermo Fisher Scientific, Rockford, IL, USA) and a chemiluminescent imaging system (FluorChem^®^ FC2; Alpha Innotech, San Leandro, CA, USA).

### Immunocytochemistry for Bax activation

Huh-7 cells were cultured on collagen-coated cover slips with Veh, 800 μM PA, 3 μM toyocamycin, or 800 μM PA + 3 μM toyocamycin for 8 h. After treatment, the cells were washed with PBS and fixed with freshly prepared 4% paraformaldehyde in PBS containing 0.1 M PIPES, 1 mM ethylene glycol-bis(β-aminoethyl ether)-N,N,N',N'-tetraacetic acid, and 3 mM MgSO_4_ at 37°C for 15 min. After a second washing step with PBS, the cells were permeabilized using 0.0125% (w/v) CHAPS in PBS at 37°C for 10 min. Next, the cells were washed with PBS and incubated in PBS containing 5% goat serum and 0.1 N NaN_3_ at room temperature for 1 h. After incubation with anti-Bax antisera (clone 6A7, 1∶400 dilution; Exalpha Biologicals, Watertown, MA, USA) at 4°C overnight, the cells were washed three times with PBS and incubated with Alexa Fluor 488-conjugated goat anti-rabbit IgG (Molecular Probes, Eugene, OR, USA) at 37°C for 1 h. All the antibodies were diluted in PBS containing 5% FBS. The cells were then washed three times in PBS and three times in water, and mounted onto slides using a ProLong^TM^ Antifade kit (Molecular Probes). The cells were imaged under an all-in-one fluorescence microscope (Biorevo BZ-9000, Keyence), employing the Z-stack function and at excitation and emission wavelengths of 488 and 507 nm, respectively. The microscope was equipped with an image analysis software program, which was used to quantify fluorescence. 6A7-Immunoreactive cells were quantified and expressed as a percentage of the total cells counted.

### Animals

The *in vivo* studies were performed in male C57BL/6J mice obtained from Kyudo Co. Ltd. All the animals were maintained at room temperature under light-controlled conditions (12 h/12 h light/dark cycle) and had free access to food and water. The animals used were 8 weeks old. The study protocol for the animal study was approved by the University of Nagasaki Animal Studies Committee. The study also followed the Guide for the Care and Use of Laboratory Animals by the National Institutes of Health. The mice were fed either a standard chow or a diet high in saturated fat, fructose, and cholesterol (FFC diet) for 4 months as previously described [25]. Male C57BL/6J mice were divided into four groups for the study (n = 36, 8–10 mice per group). The first and second groups were fed regular chow for 4 months and administered intraperitoneal injections of saline (first group)　or 0.25 mg/kg/day toyocamycin (second group) twice weekly for 2 weeks prior to sacrifice. The third and fourth groups were fed the FFC diet for 4 months. They were then administered 0.25 mg/kg/day saline (third group) or toyocamycin (fourth group) twice weekly for 2 weeks before sacrifice.

### Serum and tissue analysis

The mice were subjected to a 16-h fast before sacrifice. Under anesthesia, blood was promptly collected by cardiac puncture, which led to euthanasia. Liver tissues were then collected. Serum alanine aminotransferase (ALT), aspartate aminotransferase (AST), gELM-E]lucose, triglyceride (TG), glucose, and cholesterol levels were assessed by OREANTAL EAST Company (Shiga, Japan). Serum insulin levels were measured employing ELISA (ELM-INSULIN-1, Cosmo Bio, Tokyo, Japan) and HOMA-IR was calculated employing the following formula:fasting glucose (mg/dl) × fasting Insulin μU/ml) / 405. Whole livers were stored for later RNA analysis. Total RNA was extracted using mirVana^TM^ miRNA Isolation Kit (Ambion) according to the manufacturer’s instructions. RNA was subjected to PCR as described above. The primers for the mouse DNA are listed in Table 1.

**Table 1:** Primers for real-time PCR in mouse.

Gene	Forward primer sequence (5'-3')	Reverse primer sequence (5’-3’)
ACOX1	*CTG GGC GTA TGC CAA TTA*	*TCC AGA CTT CCA ACA TG*
DGAT1	*TCC GTC CAG GGT GGT AGT G*	*TGA ACA AAG AAT CTT GCA GAC GA*
DGAT2	*GCG CTA CTT CCG AGA CTA CTT*	*GGG CCT TAT GCC AGG AAA CT*
FASN	*GGA GGT GGT GAT AGC CGG TAT*	*TGG GTA ATC CAT AGA GCC CAG*
SCD1	*TTC TTG CGA TAC ACT CTG GTG C*	*CGG GAT TGA ATG TTC TTG TCG T*
SREBP1	*GGA GCC ATG GAT TGC ACA TT*	*GGC CCG GGA AGT CAC TGT*
18S	*CGT TCT TAG TTG GTG GAG CG*	*CGC TGA GCC AGT CAG TGT AG*

ACOX 1; Peroxisomal acyl-coenzyme A oxidase 1, DGAT1; Diglyceride acyltransferase, DGAT2; Diglyceride acyltransferase 2, FASN; Fatty acid synthase, SCD1; Stearoyl-CoA desaturase-1, SREBP1; sterol regulatory element-binding protein 1, 18S; 18S ribosomal RNA.

### Histopathology

For light microscopy (Eclipse Meta Morph V 5.0.7; Nikon, West Lafayette, IN, USA) analysis of hematoxylin and eosin-stained liver sections, liver tissue was fixed in 4% paraformaldehyde for 72 h and then embedded in paraffin. To visualize lipid droplets in the liver, the frozen liver sections were collected and were subjected to oil red O staining. The images were captured and analyzed using a fluorescence microscope (BZ-X700, Keyence).

### Statistical analysis

The data have been expressed as the mean ± standard error (SE) from at least three independent experiments. Differences between groups were compared using one-way analysis of variance (ANOVA) followed by post hoc test (using the Bonferroni method) or two-way ANOVA followed by post hoc test (using the Bonferroni method). All the tests were two-tailed and *p* values < 0.05 were considered statistically significant.

## Results

### Toyocamycin inhibits FFA-mediated steatosis and apoptosis in cultured hepatocytes

**Fig 1:**
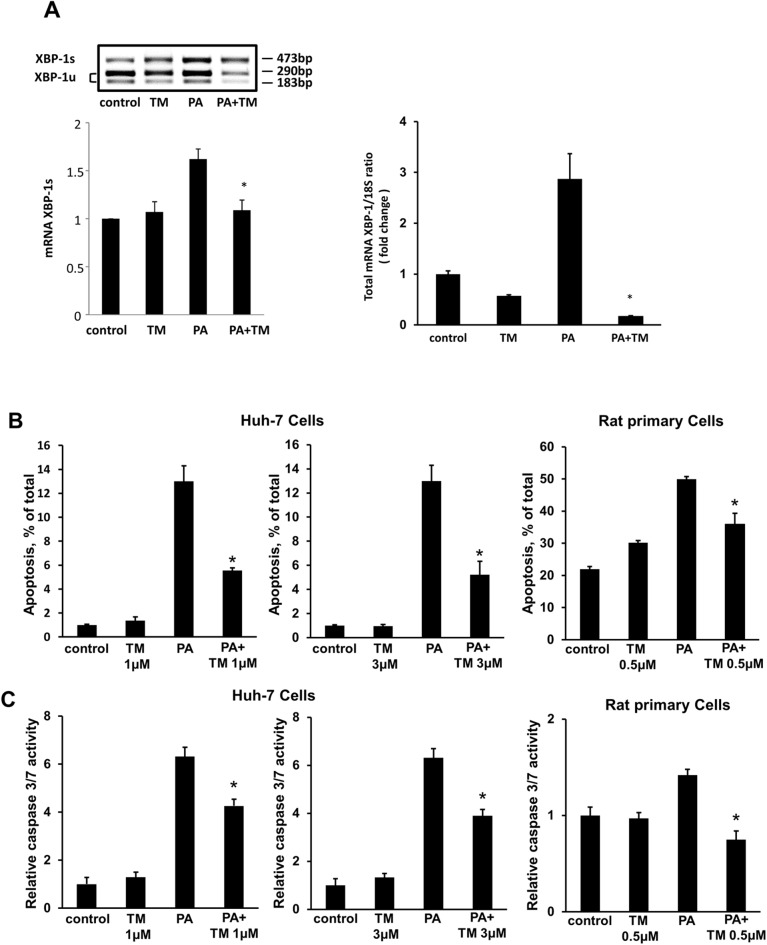
Effect of toyocamycin (TM) on palmitic acid (PA)-induced X-box binding protein-1 (XBP-1) splicing and apoptosis in cultured hepatocytes. (A) Huh-7 cells were treated with vehicle, TM (1 μM), PA (800 μM), or PA + TM for 8 h. Left upper panel: XBP-1 cDNA was amplified by real-time polymerase chain reaction (PCR) and incubated with *Pst*I for 1 h. Non-spliced XBP-1 showed 189-bp and 285-bp products, whereas spliced XBP-1 showed a 448-bp product. Left upper panel: The histogram shows quantification of the spliced XBP-1 mRNA. Right upper panel: Total XBP-1 mRNA was assessed by real-time PCR. The data are shown as fold changes relative to the values for the control group. Right lower panel: Huh-7 cells were treated with vehicle, TM (1 μM), PA (800 μM), or PA + TM for 4, 8, and 16 h. Spliced XBP-1 was assessed by immunoblotting. The data shown represent the results from three experiments. (B) Huh-7 cells were treated with PA and TM (1 or 3 μM) for 24 h. Rat primary cells were incubated with or without PA (800 μM) and TM (0.5 μM) for 12 h. (C) A caspase-3/7 assay was performed to biochemically confirm apoptosis. The data are shown as fold changes relative to the values for the control group. All data are expressed as mean ± standard error (n = 3, * indicates *P* < 0.05).

We first investigated the effect of toyocamycin on PA-induced XBP-1 mRNA expression and splicing in cultured hepatocytes. Treatment of Huh-7 cells with PA increased the levels of spliced XBP-1 m RNA; however, this effect was attenuated by toyocamycin (Fig 1A, left panel). The increase in spliced XBP-1 protein by PA was also diminished by toyocamycin (Fig 1A, right lower panel). We also observed a significant decrease in PA-induced XBP-1 mRNA expression in the Huh-7 cells treated with toyocamycin (Fig 1A, right panel).

**Fig 2:**
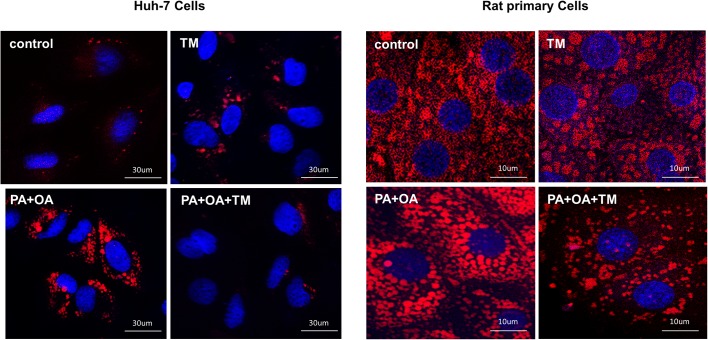
Effect of toyocamycin (TM) on free fatty acid-mediated steatosis. Vehicle-treated cells were used as controls. Huh-7 cells and primary rat cells were treated with TM (1 μM), palmitic acid (PA, 200 μM) + oleic acid (OA, 200 μM), or PA + OA + TM for 24 h. Nile red staining was performed to assess cellular steatosis. Red fluorescence shows lipids and blue fluorescence shows 4′,6-diamidino-2′-phenylindole dihydrochloride-stained sections. Signals were analyzed at a 400-fold magnification.

Furthermore, we assessed the effect of toyocamycin on lipoapoptosis. Interestingly, toyocamycin reduced PA-mediated apoptosis in both Huh-7 cells and rat primary cells (Fig 1B). Additionally, toyocamycin inhibited PA-induced apoptosis in a concentration-dependent manner as observed from the assessment of morphologic criteria (S1 Fig). Inhibition of lipoapoptosis by toyocamycin was also confirmed biochemically in the cells by evaluating caspase-3/7 activity (Fig 1C). In addition, toyocamycin significantly decreased steatosis in both vehicle-treated and FFA-treated Huh-7 cells (Fig 2). Taken together, these data indicate that toyocamycin suppressed both FFA-mediated cytotoxicity and steatosis in the cultured hepatocytes. Given that the cytoprotection conferred by toyocamycin was similar in the primary hepatocytes and Huh-7 cells, we used the Huh-7 cell line in further mechanistic assessments.

### Toyocamycin attenuates the expression of Bax-dependent DR5 and CHOP

**Fig 3:**
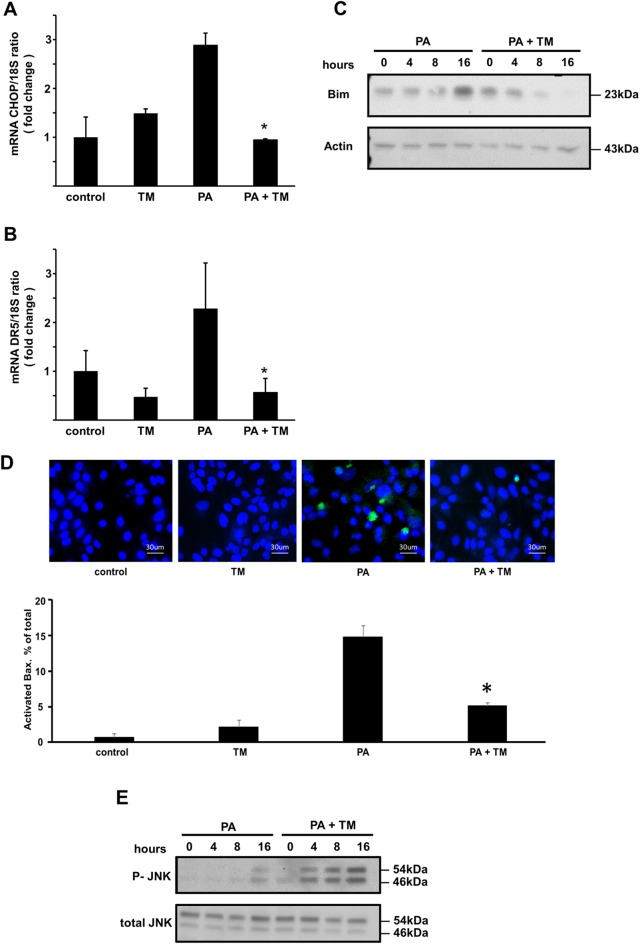
Effect of toyocamycin (TM) on the expressions of Bax-dependent Bim, DR5, and CHOP and JNK phosphorylation. (A and B) Vehicle-treated cells were used as controls. Huh-7 cells were treated with vehicle, TM (1 μM), PA (800 μM), or PA + TM for 8 h. (C) CHOP and DR5 mRNA levels were quantified by real-time polymerase chain reaction. The data are shown as fold changes relative to the values for the control group. The level of Bim was evaluated by western blot analysis. (D) Activation of Bax was examined by immunofluorescence using mouse monoclonal 6A7 Bax antibody. Green fluorescence shows activated Bax and blue fluorescence shows nucleic acids identified by 4′,6-diamidino-2′-phenylindole dihydrochloride staining. 6A7-Immunoreactive cells were quantified in 5 random × 40　objective fields for each condition. All data are expressed as mean ± standard error (n = 3). * indicates *P* < 0.05. (E) The level of total-phospho-JNK was evaluated by western blot analysis. CHOP: cytosine-cytosine-adenosine-adenosine-thymidine enhancer-binding protein homologous protein; DR5: death receptor; JNK: c-Jun N-terminal kinase.

Additionally, we explored the effect of toyocamycin on ER stress and its downstream pathways when induced by PA. We first assessed the activation of PERK response, which leads to CHOP expression. It is well accepted that PA-induced CHOP expression plays a central role in the ER stress-mediated lipoapoptotic pathway [4, 6]. PA-mediated induction of CHOP mRNA expression decreased when Huh-7 cells were coincubated with toyocamycin (Fig 3A). Cazanave *et al*. [26] previously reported that expression of CHOP stimulates the transcriptional activation of DR5, which leads to mitochondrial dysfunction. Similarly, in the present study, toyocamycin attenuated the expression of DR5 mRNA (Fig 3B) and thus, attenuated ER-stress-mediated death receptor pathways. We therefore tested the effect of toyocamycin on the proapoptotic BH3-only protein Bim during lipoapoptosis. The results showed that PA-mediated Bim protein upregulation was attenuated by toyocamycin (Fig 3C). In addition, the activation of Bax was observed by immunofluorescence using the 6A7 mouse monoclonal antibody, which only detects the active structure of Bax [4, 27]. Bax activation was observed in PA-treated cells; however, this activation decreased when the Huh-7 cells were coincubated with PA and toyocamycin (Fig 3D). Collectively, these data imply that toyocamycin blocks PA-induced ER stress and its downstream lipoapoptotic pathways mediated by mitochondria. However, toyocamycin did not attenuate the phosphorylation of JNK, which is a pathway that also is activated by IRE1α, but rather enhanced it (Fig 3E). This further confirmed that toyocamycin possibly inhibits XBP-1 in the IRE1α pathway under ER stress but does not inhibit ER stress-mediated JNK activation. This observation is consistent with the data reported in a previous study [23]. Overall, toyocamycin suppressed steatosis and ER stress-mediated apoptotic signals in Huh-7 cells despite its enhancement of JNK activation.

### Toyocamycin treatment suppresses steatosis in mice fed an FFC diet

We also tested the effect of toyocamycin in a mouse model of NAFLD [25, 28]. After 4 months of consuming an FFC-supplemented diet, the mice exhibited higher body weights than the mice fed normal chow did, which is in line with the observations in a previous study [28]. The livers of the mice showed significant steatosis and ballooned hepatocytes, as well as a minimal infiltration by inflammatory cells such as neutrophils. These findings were similar to those observed in early human NAFLD study [28]. No elevation in inflammatory cytokines was observed in the mice. In addition, no fibrosis was observed in the FFC-fed mice.

**Fig 4:**
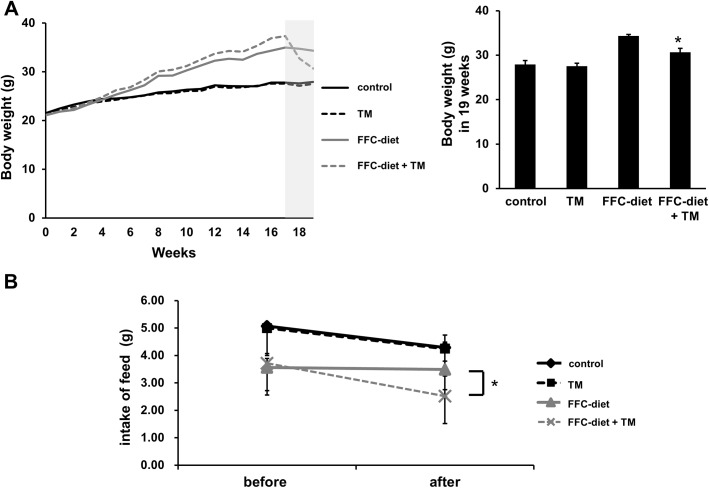
Effect of toyocamycin (TM) on the weights of saturated fat, fructose, and cholesterol (FFC) diet-fed mice. Mice were fed either normal chow or FFC diet for 4 months. Each diet group was divided into TM and saline groups. Mice in the TM group received 0.25 mg/kg/day TM twice weekly for 2 weeks. (A) Weight changes over time in mice fed standard chow (control) or FFC-diet (B) The graph shows food intake before and after TM administration.

**Fig 5:**
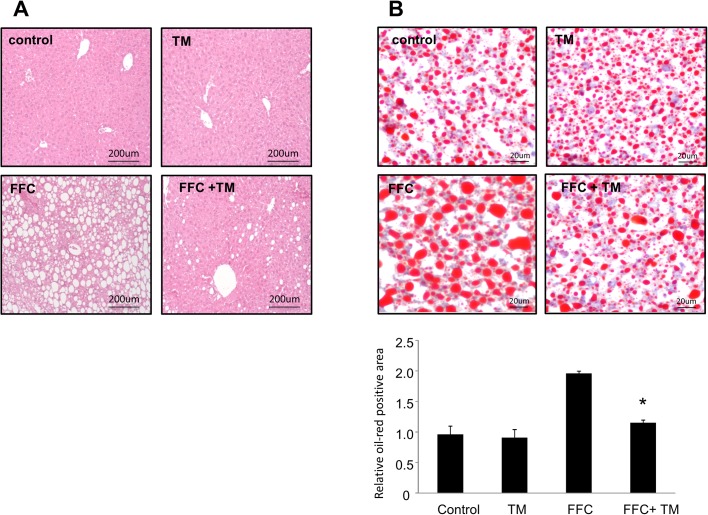
Effect of toyocamycin (TM) treatment on hepatic steatosis in mice fed a saturated fat, fructose, and cholesterol (FFC) diet. (A) Hematoxylin and eosin (original magnification, 200×) and (B) oil red O-stained sections of liver tissues (original magnification, 400×) from standard chow (control)- and FFC diet-fed mice, with and without TM treatment. The images were captured and analyzed using a fluorescence microscope (BZ-X700, Keyence). The data shown represent the results from three fields imaged from four animals from each treatment group.

**Fig 6:**
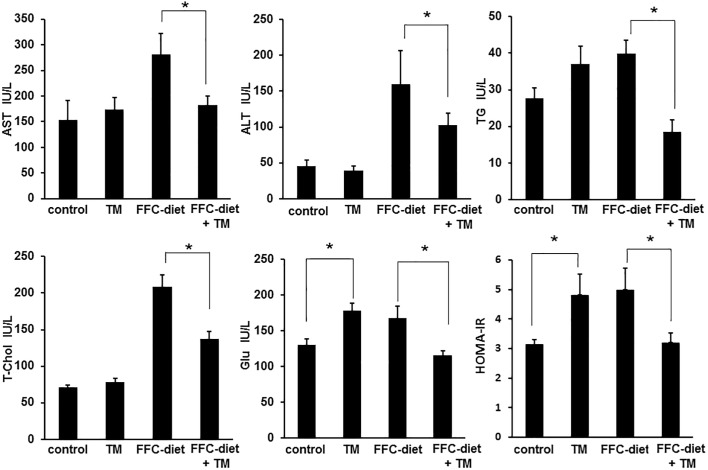
Serum levels of aspartate aminotransferase (AST), alanine aminotransferase (ALT), total cholesterol (T-cho), triglycerides (TGs), glucose (Glu), and HOMA-IR after toyocamycin (TM) administration. Mice were fed a standard chow or a saturated fat, fructose, and cholesterol (FFC) diet and administered saline or TM. Plasma AST, ALT, T-chol, TG, and Glu levels were then measured. HOMA-IR was also calculated. All the data are expressed as mean ± standard error (n = 3). * indicates *P*<0.05.

**Fig 7:**
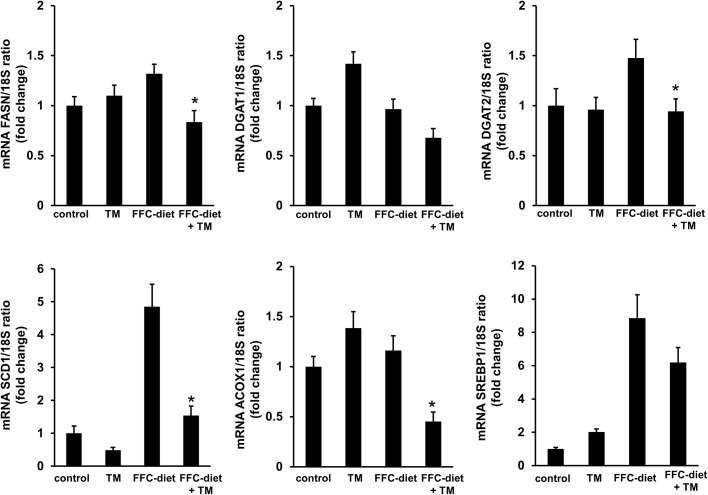
Effect of toyocamycin (TM) on the expression of lipogenic genes in mice. Mice were fed a standard chow or a saturated fat, fructose, and cholesterol (FFC) diet and administered saline or TM. FASN, SCD1, ACOX1, DGAT1, DGAT2, and SREBP1 mRNA expression levels were then measured by real-time polymerase chain reaction. Fold induction was determined by normalization to 18S. All the data are expressed as mean ± standard error (n = 3). * indicates *P* < 0.05. ACOX1: peroxisomal acyl-coenzyme A oxidase 1; DGAT: diglyceride acyltransferase; FASN: fatty acid synthase; SCD1: stearoyl-CoA desaturase-1; SREBP1: sterol regulatory element-binding protein 1; 18S: 18S rRNA.

Treatment with toyocamycin resulted in slight decreases in food intake and body weight in only the FFC-fed mice (Fig 4). Toyocamycin significantly improved hepatic steatosis as shown in Fig 5. The FFC diet also induced liver dysfunction, as revealed by elevated serum levels of ALT, AST, TG, and cholesterol. This effect was attenuated by toyocamycin (Fig 6). In FFC-fed mice, Toyocamycin attenuated serum glucose as well as homeostasis model assessment-insulin resistance (HOMA-IR) values. Unexpectedly, we noted unexpected increases in serum glucose as well as HOMA-IR in the toyocamycin treated-mice that were fed normal chow. XBP-1 has been shown to directly bind to lipogenic genes [15]. We therefore evaluated the inhibitory effect of toyocamycin on lipogenic genes. As shown in Fig 7, toyocamycin attenuated the mRNA expressions of FAS, SCD-1, ACOX, and DGAT2 in the FFC-diet fed mice. Although toyocamycin tended to decrease the expression levels of SREBP and DGAT1 mRNAs, the observed differences were not statistically significant (Fig 7). The data obtained implied that the treatment with toyocamycin ameliorated the adverse effects of the FFC diet-induced NAFLD in the mice, at least partly by suppressing lipogenic gene expression.

## Discussion

The major findings of this study were as follows. Toyocamycin suppressed FFA-induced apoptotic pathways as well as steatosis in the cultured hepatocytes. It also decreased serum liver dysfunction indicators and the serum levels of total cholesterol and TG in the FFC-fed mice. Lastly, it decreased steatosis and the expression of lipogenic genes in the FFC-fed mice.

Lipotoxicity is a complicated process encompassing multiple types of cellular dysfunction. In particular, the signaling pathways involving ER stress, JNK, and death receptor 5 (DR5) are key components in PA-induced apoptosis. Our study showed that toyocamycin attenuated lipotoxicity and the expression of CHOP and DR5. *In vitro*, CHOP accelerated PA-induced lipoapoptosis in hepatocytes, whereas XBP-1 was found to either directly or indirectly inhibit CHOP, thereby decreasing lipoapoptosis. Although it has been reported that XBP-1 can bind to the CHOP promoter, the functional effect of this phenomenon has not been elucidated. In addition, we speculate that the antilipoapoptotic effect of XBP-1 was partially achieved by reducing FFA levels within the hepatocytes.

Our study also indicated that toyocamycin suppressed lipoapoptosis despite its enhancement of JNK activity. JNK can be involved in either cell survival or cell death [29, 30]. Although JNK is considered to play a pivotal role in lipoapoptosis, a recent study revealed that the activation of JNK is ER stress-independent. Rather, it has been suggested that GSK3, a serine/threonine protein kinase, and MLK3 directly activate JNK via phosphorylation and thus induce apoptosis [8, 9]. Activation of JNK in the presence of toyocamycin presumably results from a negative feedback caused by the inactivation of XBP-1 splicing in the IRE1α pathway under ER stress [23]. Thus, we considered the possibility that ER stress-induced JNK activation may not have cytotoxic effects on hepatocytes.

In our study, neither did toyocamycin show toxicity against isolated hepatocytes or Huh-7 cells, nor did it induce hepatotoxicity when injected into the mice. Toyocamycin was initially described as a potential anticancer drug. Indeed, several types of tumor cells including multiple myeloma cells and leukemia cells are sensitive to toyocamycin [23, 31, 32]. We also found that toyocamycin was toxic to HepG2 and Hep3B cells (data not shown). Interestingly, total XBP-1 knockout mice are embryonically lethal; thus, XBP-1 appears to be crucial for fetal development [33]. In contrast, specific inducible deletion of XBP-1 in the adult mouse liver results in reduced *de novo* lipogenesis without inducing evident ER stress [15, 34]. The reasons for the aforementioned differences in sensitivity to XBP-1 inhibition are unclear. It has been suggested that some cell types, such as those involved in the development of cancer cells, may require XBP-1 for survival. However, under certain conditions, such as those present during NAFLD, XBP-1 mainly plays a role in lipogenesis.

Elevated levels of saturated fatty acids in the blood are considered important for inducing steatosis. Lipogenesis from glucose is considered an important contributing factor for steatosis and NASH. High carbohydrate diets, including fructose diets, increase *de novo* lipid synthesis in the liver by inducing the expression of genes that encode lipogenic enzymes [15, 35]. FFC diet-fed animals with elevated serum levels of saturated FFAs and sugar components have been recognized to present NAFLD characteristics with similarities to the human condition [25]. XBP-1 expression is induced in the liver by a high carbohydrate diet, as well as by FFA-rich diets. XBP-1 is thought to induce the expression of a set of critical genes involved in fatty acid synthesis [15, 34]. Inducible deletion of XBP-1 in the adult liver results in hypocholesterolemia and hypotriglyceridemia, as well as a decreased production of lipids in the liver [15, 34]. In line with these findings, the current study showed that toyocamycin suppressed FFC-induced hepatic production of lipids, TGs, and cholesterol. Neutral lipid accumulation in the liver is associated with an enhanced expression of lipogenic genes, including acetyl-CoA carboxylases (ACCs), fatty acid synthase (FAS), and stearoyl-CoA desaturase-1 (SCD1). These enzymes are mainly regulated by sterol regulatory element-binding protein (SREBP) [36]. Although the expression of SREBP1 tended to decrease during the toyocamycin treatment in the present study, the decrease was not statistically significant. These findings are similar to those of Lee et al. [15], who used an inducible knockdown of XBP-1 for their study. Because spliced XBP-1 can directly bind to the promoter regions of Dgat2, Scd1, and Acc2, toyocamycin may be able to inhibit these genes equally by inactivating XBP-1. In contrast, Liu et al. [21] studied hepatocyte-specific Xbp-1-deficient (XBP-1 -/-) mice and found that genetic deletion of XBP-1 enhanced liver injury. The reason for this outcome is unclear; however, we believe that the effect of deleting XBP-1 in a fetus may be different from that in the adult organism.

Toyocamycin may have an unknown role aside its inhibition of XBP-1 in hepatocytes. For example, it has been shown that toyocamycin inhibits the activities of various kinases, including protein kinase C, in myelogenous leukemia cells [37]. A recent study suggested that toyocamycin specifically inhibits the splicing of XBP-1 [23]. However, the results of our study in the hepatocytes suggest that it also decreases the total XBP-1 mRNA level in hepatocytes, which could have contributed to the reduced amount of active XBP-1 we observed.

Our studies demonstrated that Toyocamycin attenuated liver dysfunction as well as serum TG and cholesterol levels in FFC-fed mice. Interestingly, although toyocamycin treatment improved insulin resistance in the mice with regard to metabolic syndrome, we noted that serum glucose and HOMA-IR values increased in the lean mice after the treatment. Thus, toyocamycin may have two distinct effects in glucose metabolism. It has the potential to cause metabolic disturbance in lean mice although it does not induce liver toxicity. In the FFC-fed mice, it improved the outcome of NAFLD, probably by the multifactorial pathways listed above. We consider that toyocamycin shows the effects against NAFLD at least, in part, by acting directly in the liver. Furthermore, we observed reductions in body weight and food intake in the toyocamycin-treated mice that were fed the FFC diet. In addition to directly reducing hepatic lipid accumulation, toyocamycin may have attenuated steatosis and liver dysfunction by some other distinct mechanisms. Although we did not perform any direct studies on peripheral tissues in the present study, it appears that the reversal of steatosis by toyocamycin may have resulted from its dramatic effect in inhibiting lipogenesis in peripheral tissues. Toyocamycin may have also exerted unknown effects on the mice, such as loss of appetite, that were independent of XBP-1 inhibition. Nevertheless, we noted that the reduced food intake was only observed in the FFC diet-fed mice. One of the limitations of our study was that we used a mouse model that replicated early-stage NAFLD, which did not allow us to assess the effect of toyocamycin in severe liver injury and/or fibrosis. The mechanism underlying toyocamycin-induced XBP-1 inhibition in NAFLD/NASH remains to be clarified; however, such a study may be complicated by the presence of complex metabolic interactions in the liver. A novel potential role of XBP-1 in the development of NAFLD/NASH was recently described [38]. As hepatocytes die, they release extracellular vesicles containing FFAs; however, that pathway is IRE1α-XBP-1 signal-dependent and contributes to inflammation [38]. Rivera et al. [39] reported that adenovirus-mediated expression of dominant negative XBP-1 led to significant attenuation of high fat, high cholesterol diet-induced liver inflammation in mice. The liver　inflammation was likely mediated by Toll-like receptor-4, which is a proinflammatory mediator that is associated with hepatic injury and fibrosis [39]. Thus, inhibition of XBP-1 by toyocamycin has the potential to inhibit inflammatory responses during elevations in serum FFA levels. However, this mechanism requires further investigations employing a more severe model of NASH.

In conclusion, our data show that toyocamycin suppressed FFC diet-induced liver injury and steatosis in mice, as well as PA-induced hepatocyte lipoapoptosis. These results suggest that compounds that inhibit XBP-1 may be beneficial for treating NAFLD and other metabolic diseases.

## Supporting information

S1 Fig Toyocamycin inhibits FFA-mediated apoptosis in a concentration-dependent manner in Huh-7 cells. Huh-7 cells were treated with either vehicle, palmitic acid (PA, 800 μM), or PA plus toyocamycin (0.1 μM, 0.5 μM, 1 μM, or 3 μM) for 24 h. The targets were displayed as fold changes relative to the control. All data are the mean±SE for 3 experiments. *P<0.05.
(TIF)
