# Correction: Toyocamycin attenuates free fatty acid-induced hepatic steatosis and apoptosis in cultured hepatocytes and ameliorates nonalcoholic fatty liver disease in mice

**DOI:** 10.1371/journal.pone.0235729

**Published:** 2020-07-01

**Authors:** Ikuko Takahara, Yuko Akazawa, Maiko Tabuchi, Katsuya Matsuda, Hisamitsu Miyaaki, Youko Kido, Yasuko Kanda, Naota Taura, Ken Ohnita, Fuminao Takeshima, Yusuke Sakai, Susumu Eguchi, Masahiro Nakashima, Kazuhiko Nakao

There is an error in affiliation 1 for authors Ikuko Takahara, Yuko Akazawa, Maiko Tabuchi, Hisamitsu Miyaaki, Youko Kido, Yasuko Kanda, Naota Taura, Ken Ohnita, Fuminao Takeshima, and Kazuhiko Nakao. The correct affiliation 1 is: Department of Gastroenterology and Hepatology, Nagasaki University Graduate School of Biomedical Sciences, Nagasaki, Japan.
