# Supplementary figures and images for: Toyocamycin attenuates free fatty acid-induced hepatic steatosis and apoptosis in cultured hepatocytes and ameliorates nonalcoholic fatty liver disease in mice
Source: PLoS One. 2017 Mar 9;12(3):e0170591. doi: 10.1371/journal.pone.0170591 (PMC5344317; doi:10.1371/journal.pone.0170591)

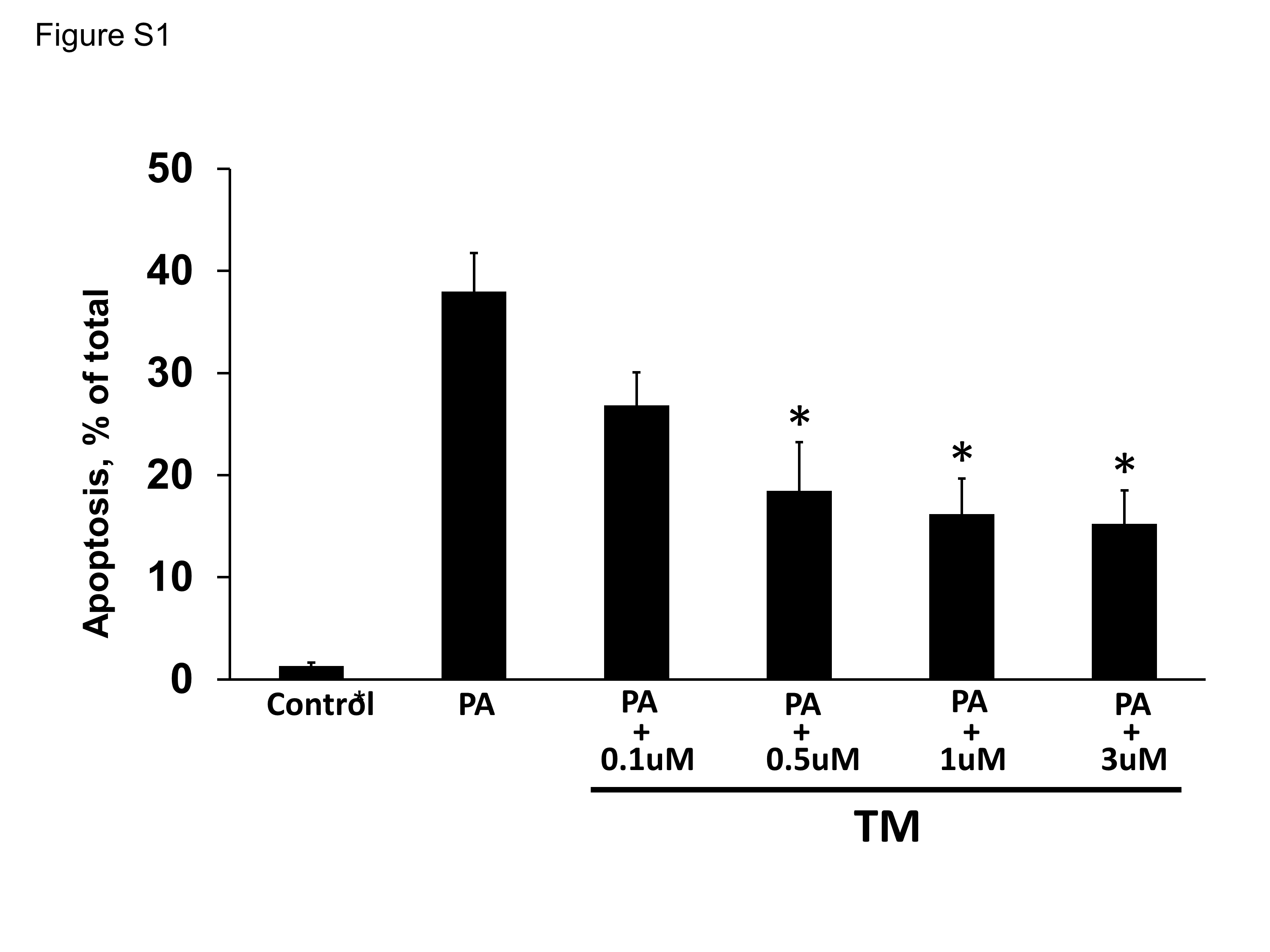

Supplement: S1 Fig — Huh-7 cells were treated with either vehicle, palmitic acid (PA, 800 μM), or PA plus toyocamycin (0.1 μM, 0.5 μM, 1 μM, or 3 μM) for 24 h. The targets were displayed as fold changes relative to the control. All data are the mean±SE for 3 experiments. *P<0.05. (TIF) [file pone.0170591.s001.tif]
